# Domain-Specific Self-Reported and Objectively Measured Physical Activity in Children

**DOI:** 10.3390/ijerph14030242

**Published:** 2017-03-01

**Authors:** Ole Sprengeler, Norman Wirsik, Antje Hebestreit, Diana Herrmann, Wolfgang Ahrens

**Affiliations:** 1 Department of Epidemiological Methods and Etiologic Research, Leibniz Institute for Prevention Research and Epidemiology—BIPS, Achterstraße 30, 28359 Bremen, Germany; sprengel@leibniz-bips.de (O.S.); hebestr@leibniz-bips.de (A.H.); Diana.Herrmann@dguv.de (D.H.); 2 Department of Biometry and Data Management, Leibniz Institute for Prevention Research and Epidemiology—BIPS, Achterstraße 30, 28359 Bremen, Germany; wirsik@leibniz-bips.de; 3 Institute of Statistics, Faculty of Mathematics and Computer Science, University Bremen, 28359 Bremen, Germany

**Keywords:** accelerometry, exercise, intermethod reliability, physical activity questionnaire, youth

## Abstract

Little is known about the extent that different domains contribute to total sedentary (SED), light (LPA) and moderate-to-vigorous physical activity (MVPA). We aimed to identify domain-specific physical activity (PA) patterns in school-aged children who were assessed by questionnaire and accelerometry. For the study, 298 German school children and adolescents aged 6–17 years wore an accelerometer for one week and completed a PA recall-questionnaire for the same period. Spearman coefficients (r) were used to evaluate the agreement between self-reported and objectively measured PA in five domains (transport, school hours, physical education, leisure-time, organized sports activities). School hours mainly contributed to the total objectively measured SED, LPA and MVPA (55%, 53% and 46%, respectively), whilst sports activities contributed only 24% to total MVPA. Compared to accelerometry, the proportion of self-reported LPA and MVPA during school hours was substantially underestimated but overestimated during leisure-time. The agreement of self-reported and objectively measured PA was low for total LPA (r = 0.09, 95% CI (confidence interval): −0.03–0.20) and total MVPA (r = 0.21, 95% CI: 0.10–0.32), while moderate agreement was only found for total SED (r = 0.44, 95% CI: 0.34–0.53), LPA during transport (r = 0.59; 95% CI: 0.49–0.67) and MVPA during organized sports activities (r = 0.54; 95% CI: 0.38–0.67). Since school hours mainly contribute to total SED, LPA and MVPA and self-reported LPA and MVPA during school were importantly underestimated compared to objectively measured LPA and MVPA, the application of objective measurements is compulsory to characterize the entire activity pattern of school-aged children.

## 1. Introduction

Physical activity (PA) is known to be beneficial for human mental and physical health. In children and adolescents, 60 min of moderate-to-vigorous PA (MVPA) per day are recommended because these were associated with lower cardiovascular disease risk, higher fitness levels and improved bone strength [1,2,3]. Nowadays, less than a third of all children and adolescents do not meet the current PA guidelines [4,5]. The association of sedentary behaviour (SED) with metabolic diseases has been increasingly studied in recent years [6], with particular attention being paid to children and adolescents [7]. SED may occupy increasing amounts of young people’s time, as the number, variety, availability and attractiveness of electronic media is increasing [7]. According to the SLOTH (sleep, leisure, occupation, transportation, household) model by Pratt and colleagues, opportunities for children and adolescents to accumulate PA can be assigned to five domains: sleep, transportation, school time, leisure time and home [8]. In particular, the school offers a major opportunity for children and adolescents to be physically active as this is where they spend about half of their waking hours [6,9,10].

In epidemiological studies, PA and SED are usually assessed using subjective methods by asking children or their parents to report the duration of outdoor playing time, organized sports activities or electronic media consumption [5]. These domain-specific PA variables are assumed to reasonably characterize youth’s activity behaviour [8,11]. Although single activity behaviours like commuting, playing outdoors, and organized sports activities are associated with objectively measured MVPA [12,13], the correlation of subjectively and objectively measured PA is usually low or at best moderate [14,15].

Thus, researchers usually have to weigh the validity against the feasibility of the available PA and SED assessment methods, particularly in population-based field studies. To collect PA data in large samples, questionnaires are the most feasible method, but, typically, they tend to overestimate PA and SED [16]. Since different intensities such as MVPA and SED occur during daily activities throughout the whole day, all intensities are prone to misreporting by self- and proxy-reports. In contrast, accelerometry has been shown to be a valid and reliable method to estimate levels of PA and SED [17], their mere measurement lacks to assess the context in which they occur. Accordingly, the combined application of subjective and objective methods is recommended [11,15,16,18,19]. Up to now, studies investigating which domains contribute most to total sedentary (SED), light PA (LPA) and MVPA are rare [6,20].

In order to characterize youth’s PA behaviour and to develop and implement successful interventions [12,21], domains that support longer duration of PA and help to decrease SED have to be identified [15,22]. The present study aimed to investigate the contribution of four different domains (transportation, school time, physical education, leisure time and organized sports activities) to total SED, LPA and MVPA, assessed by accelerometry and questionnaires. Furthermore, we aimed to evaluate the agreement of self-reported and objectively measured PA in order to determine potential under- and overestimations that might result when questionnaires are used to characterize school children’s PA.

## 2. Materials and Methods

### 2.1. Study Sample

This cross-sectional study was carried out in two primary and two secondary schools in the city of Bremen, Germany, between September 2012 and February 2013. The participating schools (two of them located in a middle-income area and two in a high-income area) were selected to cover a broad spectrum of socioeconomic groups in each age category. The schools were initially approached through phone calls, and, after an invitation by the principal of each school, the study was presented to the staff personally by trained study personnel. A total of 335 children (6–10 years) from 14 classes and 207 adolescents (11–17 years) from 10 classes were invited to participate in the study, and their parents were asked for written informed consent. Adolescents (11–17 years) were also asked for written consent, while younger pupils (6–10 years) were also asked for oral consent. The study was conducted in accordance with the Declaration of Helsinki, and the protocol was approved by the institutional review board of the University of Bremen (06-3, approved on the 18 November 2011).

### 2.2. Data Collection

Subjective (7-day recall PA questionnaire, accelerometer diary) and an objective (7-day-accelerometry) PA assessment methods were deployed in parallel to cover the same period. Subjects were asked to complete the PA questionnaire (PAQ) for the previous seven days on the last day of the accelerometer wearing period. For the younger pupils (6–10 years), the times when school started and ended for each schoolday on which they wore an accelerometer were provided by the teachers. Finally, body-mass-index (BMI, kg/m^2^) was calculated based on self-reported height (m) and weight (kg).

#### 2.2.1. Accelerometry

PA and SED were measured using Actigraph accelerometers (GT3X+, GT1M, ActiTrainer; Pensacola, FL, USA) for seven consecutive days, including weekdays and weekend days. PA data were recorded at three-second epochs and computed with the ActiLife 6 software (ActiGraph, Pensacola, FL, USA). Although we applied 3 different Actigraph accelerometers, these devices have been proven to provide comparable PA data [23,24,25,26]. The accelerometers were attached with an elastic belt and worn on the right hip for seven consecutive days during waking hours, except when showering, bathing and swimming. Accelerometer data were only included when PA was recorded for a minimum of three days and 10 h per day. Periods with consecutive zero counts of 90 min and more were excluded from the analysis [27]. To distinguish between different PA intensities, accelerometer counts were categorized according to the cut-off points proposed by Evenson and colleagues (SED: ≤100 cpm, LPA: >100<2296 cpm, and MVPA: ≥2296 cpm) [28].

#### 2.2.2. Accelerometer Diary

All participants were asked to complete a short diary to record times of going to bed and waking up in the morning, times of sports activities (physical education classes and organized sports activities) as well as reasons of non-wear-time. The participants were instructed verbally and in writing on how to use the accelerometer and the diary.

#### 2.2.3. Seven-Day Recall PA Questionnaire (PAQ)

We used a modified version of the validated German MoMo-(Motoric Module) PAQ to assess PA [29]. The modified PAQ consists of 12 questions assessing five domains of PA (transport, school time, physical education, leisure time and organized sports activities) for seven consecutive days. Children up to the age of 10 were advised to complete the questionnaire together with their parents (proxy-reports), while the older students were asked to complete the questionnaire by themselves. Subjects were asked about frequency, duration and intensity of PA for each domain. In particular, the intensity was assessed by asking participants about their self-perceived, typical intensity of breathing and sweating during PA. They were asked to choose one of the following intensity categories: “without sweating and getting out of breath”, “sweating and getting out of breath a little bit” and “sweating and getting out of breath a lot”, which were assigned to the PA intensity level—LPA, moderate PA (MPA) and VPA, respectively. Minutes in MPA and VPA were then combined to MVPA. SED was measured by asking participants about the duration spent sitting during transport, school during leisure time. While participants were asked to assign a typical intensity level to recurrent activities like active transport, physical education and organized sports activities, they were asked to specify the duration in each intensity category for irregular activities during school and leisure time (e.g., walking, running). Finally, the reported time (minutes) across all activities was summed up by domain and intensity category.

We allowed up to four questions to be skipped because not all enquired activities applied to each child. Accordingly, self-reported PA data were considered plausible when at least 67% of all 12 questions were completed. Extreme values—defined as lying outside the interval (first quartile − 1.5 × interquartile range, third quartile + 1.5 × interquartile range)—were considered as outliers and excluded from the analysis [30]. Additionally, all reported time data were proportionally scaled so that the cumulative duration of SED, LPA and MVPA over all domains agreed with the reported waking hours (time between getting up in the morning and going to bed at night). A scaling factor was calculated as waking hours divided by the sum of reported PA across all domains. Reported times in the domains was then multiplied by the scaling factor, so that of reported PA matched the time awake. The scaling factor was calculated only after subtracting the duration of transport, physical education and organized sports activities, and these activities were not scaled, as it is plausible that these domains were reported quite accurately.

#### 2.2.4. Time-Stamping of PA Data

Originally, the SLOTH model assesses the following five domains of PA: sleep, leisure, occupation, transportation and household [8]. In order to investigate the contribution of potential high-intensity subdomains of PA, we adapted the model by excluding the domains’ sleep and household and by including physical education as a subdomain of school time and by including organized sports activities as a subdomain of leisure time. The start and end time of school was provided by teachers in the form of schedules.

Based on the accelerometer diary, the PAQ and the schedules, self-reported and objectively measured SED, LPA and MVPA were each assigned to the domains’ transport, school time, physical education, leisure time and organized sports activities. The times of physical education and organized sports activities reported in the activity diary were each aligned with the accelerometer data. School time was defined as the interval between the start and end of each school day, subtracting physical education lessons. The exact time frames of transport were not recorded in the diary, but the duration of transport in the questionnaire. In order to identify the domain transport, we took the start and end time of school and subtracted, and respectively added the reported duration to these times plus an additional 5 min to add a margin, assuming that students do not arrive just in time for school and leave right away. As PA during transport, school time and physical education were not applicable during weekend days, time intervals were classified as leisure time and organized sports activities.

The sum of all domain-specific time intervals in SED, LPA and MVPA per day was divided by the number of valid weekdays and weekend days to obtain average PA levels on a daily basis. Weekdays and weekend days were analyzed separately. The duration of SED, LPA and MVPA for the domains’ transport, school and leisure time was expressed in minutes per day, whereas times of physical education and organized sports activities were expressed in minutes per week. To calculate the daily proportion of PA in the different domains, times of physical education and organized sports activities were also converted to minutes per day (total duration/number of days).

#### 2.2.5. Analysis Group

**Figure 1:**
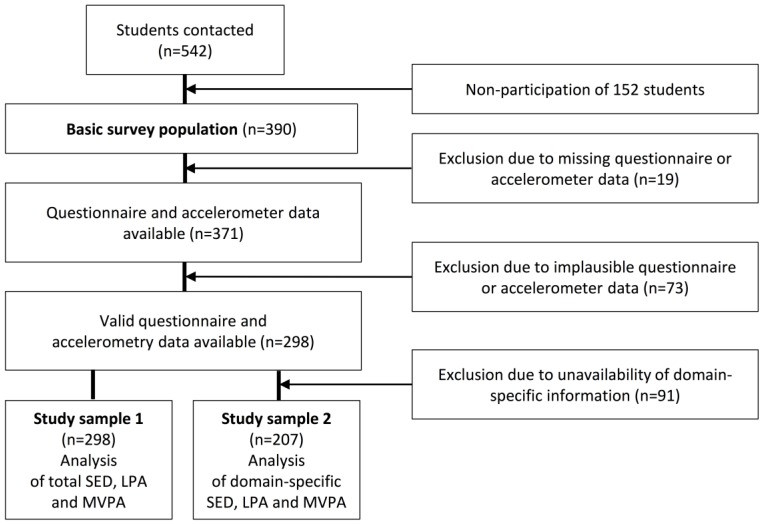
Number of included and excluded participants of the study population. SED = sedentary behaviour; LPA = light physical activity; MVPA = moderate-to-vigorous physical activity.

In this study, 542 children and adolescents were invited to participate. Informed consent was obtained from 390 (72%). Though written consent of the parents was obtained, 2 pupils denied to participate in the study. The study sample was divided into two groups because domain-specific information was not available for all children. Figure 1 shows the composition of the study sample and the exclusion criteria. Accelerometer and questionnaire data were provided by 371 participants.

Study sample 1 includes all participants aged 6–17 years (*n* = 298, 52.7% boys) having valid self-reported and objectively measured PA data (at least two weekdays and one weekend day). Since reliable domain-specific information on the start and end of school in the form of schedules was only provided by teachers of children aged 6–10 years (*n* = 207, 49.8% boys), the domain-specific analysis of SED, LPA and MVPA could only be conducted for study sample 2 with valid self-reported and objectively measured PA data for at least 3 weekdays (*n* = 207).

### 2.3. Statistical Analysis

Self-reported and objectively measured minutes of total and domain-specific SED, LPA and MVPA were compared using the Spearman rank correlation coefficient r_S_. The correlation is considered as weak if r_S_ ≤ 0.39, as moderate if r_S_ = 0.40–0.59, as strong if r_S_ = 0.60–0.79 and as very strong to perfect if r_S_ ≥ 0.80. To assess the agreement of self-reported MVPA vs. objectively measured MVPA with regard to the WHO recommendation, Cohen’s kappa coefficient κ was calculated. κ has the same range as r_S_ and the same interpretation of the strength of agreement [31]. Additionally, two sensitivity analyses were conducted to investigate the impact of (a) scaling the total reported PA according the reported time awake and (b) different definitions of valid self-reported PA data (67% vs. 83% completed questions). The level of statistical significance was set at α = 0.05. Correlation coefficients were reported with 95% confidence interval (95% CI). All analyses were performed with SAS 9.3 (SAS Institute Inc., Cary, NC, USA).

## 3. Results

### 3.1. Descriptive Values

Mean values and standard deviation (SD) of demographic, anthropometric and PA variables are shown in Table 1. Self-reported and objectively measured SED, LPA and MVPA were analyzed for 207 children aged 6–10 years and 91 adolescents aged 11–17 years (Table 1). Daily and overall accelerometer wear-time was similar in children and adolescents and in boys and girls. In both age groups, self-reported SED was lower than objectively measured SED. SED was lower in children aged 6–10 years compared with adolescents. Generally, self-reported LPA (193 min/day) and MVPA (116 min/day) were higher than objectively measured LPA (163 min/day) and MVPA (65 min/day). This difference was more pronounced in adolescents aged 11–17 years. The daily duration of MVPA was generally higher in boys than in girls. Both self-reported and objectively measured MVPA were higher in children aged 6–10 years (118 ± 78 min/day and 75 ± 20 min/day, respectively) than in adolescents aged 11–17 years (113 ± 94 min/day and 56 ± 21 min/day, respectively). Accordingly, more children aged 6–10 years than adolescents aged 11–17 years achieved the WHO PA recommendations of at least 60 min of MVPA per day: 70% according to self-reported data and 62% according to accelerometry, as compared to 61% and 36%, respectively. A considerably larger proportion of all participants achieved the recommended PA levels on weekdays than on weekend days.

### 3.2. Duration and Proportion of SED, LPA and MVPA in Different Domains

Table 2 shows that school and leisure time contributed most to SED and LPA. Compared with accelerometry, MVPA was underreported for transport and school time and overreported for physical education and leisure time, while LPA was overreported for transport and leisure time and underreported for school time, sports lessons and sports activities. SED was generally underreported except for sports activities. Together, objectively measured MVPA during physical education and during organized sports activities contributed 24% to total MVPA, whilst self-reported MVPA in these two domains accounted for twice as much of total MVPA (48%). Based on accelerometry, school time, leisure time and organized sports activities contributed most to total MVPA (46%, 23% and 16%, respectively) and to total SED (55%).

### 3.3. Correlation of Self-Reported and Objectively Measured Total SED, LPA and MVPA

The correlation between self-reported and objectively measured SED, LPA and MVPA was generally weak (Table 3). The best correlation was observed for SED (children: r = 0.28, 95% confidence interval (CI) = 0.15–0.40; adolescents: r = 0.35, 95% CI = 0.15–0.52). Smaller correlation coefficients and wider CIs were observed for MVPA. The correlation was negligibly small for LPA. Using the PA recommendations of at least 60 min MVPA per day as a cut-off, κ coefficients indicated no agreement in children aged 6–10 years and weak agreement in adolescents aged 11–17 years.

### 3.4. Domain-Specific Correlation for SED, LPA and MVPA

The domain-specific correlations between self-reported and objectively measured SED, LPA and MVPA were generally weak or absent with two exceptions: moderate correlations were observed for the duration of MVPA during organized sports activities (r = 0.54; 95% CI: 0.38–0.67) and for the duration of LPA during transport (r = 0.59; 95% CI: 0.49–0.67) (see Table 4).

To control if the applied cut-off for a valid questionnaire (completion of at least eight out of 12 questions) does not lead to misinterpretations, a sensitivity analyses was conducted. After raising the cut-off for inclusion of up to 10 questions, no substantial differences of total and domain-specific SED, LPA and MVPA were observed.

## 4. Discussion

School time was the main contributor to total objectively measured SED, LPA and MVPA. The agreement of self-reported and objectively measured PA and SED was generally low, with the exception of MVPA during organized sports activities and LPA during transport. Compared to accelerometry, self-reported LPA and MVPA during school hours were substantially underestimated. All in all, our results confirm the limited validity of internationally applied PA questionnaires [15]. To our knowledge, this is the first study assessing self-reported and objectively measured proportions of SED, LPA and MVPA in the domains’ transport, school time, physical education, leisure time and organized sports activities in children aged 6–10 years.

### 4.1. Differences of Age- and Sex-Specific PA

The observed downward trend of MVPA with increasing age in schoolchildren is corroborated by previous studies [6,12] as is the higher amount of total MVPA in boys as compared to girls [5,6,10,20], especially during physical education [32]. Moreover, boys spent less time sedentary during school days than girls [6,20].

The proportion of children who were regularly active at organized sports activities in our study (63%) was similar to that reported in a survey representative of German children and adolescents (75%), the KIGGS (German Health Interview and Examination Survey for Children and Adolescents) study [33]. Total and domain-specific correlations of self-reported and objectively measured MVPA were reported for children aged 9 to 17 years in a subsample, the MoMo study (18) observing similarly weak correlations for total MVPA as our study (r = 0.29 and r = 0.21, respectively). Similar to our results, boys were more active than girls. However, there were some differences between our findings and those of the MoMo study. The observed average duration of objectively measured (39 min) and self-reported (89 min) MVPA were both lower than in our study (56 min and 113 min, respectively). This may be due to the younger children included in our study, who typically tend to be more active than older children [12,34]. The domain-specific correlations observed in the MoMo study were at best low (r = 0.04 to 0.35), with the best agreement observed for organized sports activities. In contrast, we observed moderate agreement during transport and organized sports activities. Apparently, better correlations are observed in domains that refer to regular and well-defined activities. Such activities may be easier to recall for respondents. A possible explanation why these correlations were found higher than in the MoMo study may be due to the shorter sampling interval of 3 s used in our study that is known to be more accurate than longer sampling intervals [35].

Self-reported LPA and MVPA during school time were underreported compared to accelerometry in our study. In younger children aged 6–10 years, this finding could be attributed to the proxy reporting. However, adolescents aged 11–17 years did not show better agreement. We conclude that questionnaires cannot assess total PA levels with acceptable accuracy. Nevertheless, self-reports provide information on context and type of PA and may thus be a useful complement to objective measurements.

### 4.2. Differences of Domain-Specific PA

By combining GPS monitors and accelerometry, researchers in the past were able to investigate the context of PA without using subjective methods [6,21,36]. These studies, however, came to different conclusions. While Bailey and colleagues reported the highest proportions of daily SED, LPA and MVPA to accumulate during school time (63.9%) and leisure time (25.8%) in children aged 10–14 years from the UK [6], Rainham and colleagues measured the highest proportion of MVPA during commuting in Scottish adolescents aged 12–16 years [21]. Particularly when physical education is added to school time and when organized sports activities are added to leisure time, the contribution of school and leisure time becomes 54% and 39%, respectively. Hence, our findings are in good agreement with those of Bailey and colleagues, but not with those of Rainham and colleagues. The latter concluded that transport contributes to a similar extent to total MVPA as home and school time in 12- to 16-year old adolescents. In particular, adolescents living in urban and suburban areas of Halifax accumulated most of their MVPA during transport. In contrast, school time contributed mostly to total MVPA in adolescents living in rural areas [21]. Indeed, the study population of Rainham and colleagues was apparently older than in our study and the domain transport involved all commuting activities between locations, not solely commuting to school.

In comparison to school or leisure time, physical education and organized sports activities contribute to a lesser extent to total MVPA (8% and 16%, respectively). According to Klinker and colleagues, these domains contribute to a lesser extent to total MVPA because they are not visited daily [36]. Guinhouya and colleagues reported that recess at school accounted for up to 70% of total MVPA per day, although they only included school days without structured PA (i.e., physical education) [10].

In our study, the contribution of the domain school to total SED, LPA and MVPA was significantly influenced by the duration of school time. Most children in our study spent 8 h per day at school (*n* = 182), which is not unusual in European primary schools [10,20]. In comparison to children who spent 5.5 h per day at school (*n* = 25), they accumulated 15%–20% more SED, LPA and MVPA during school time. Until now, evidence is scarce as to how the duration of school time impacts PA levels. Future studies should investigate to what extent school characteristics vary between countries and in different age groups.

In contrast to other studies [6,32], we did not observe substantial differences of domain-specific SED, LPA and MVPA between boys and girls. These contradictory findings may be explained by the inclusion of younger children in our study, who generally are found to be more active independent of their sex [12,34].

### 4.3. Strengths and Limitations

The major strength of this study is the simultaneous application of subjective and objective methods to assess all domains and intensities of PA [15,18,19] and to overcome the limitations of each method [11,15,16,19,37]. Both subjective and objective methods applied in this study were previously validated [26,29]. The Actigraph accelerometers are recommended devices to measure SED, LPA and MVPA in children and adolescents [18,26,35,38]. Moreover, to provide valid objective PA data by accelerometry, we achieved the recommended wearing times on at least four weekdays and one weekend day (average wear-time: 13.4 h/day on 4.5 weekdays and 12.4 h/day on 1.6 weekend days, respectively) [39]. The combined use of the diary and accelerometer enabled the time-stamping of accelerometer data and the assignment of objectively measured SED, LPA and MVPA to different domains. For instance, the adaption of the SLOTH model (considering the subdomains’ physical education and organized sports activities) facilitated the investigation of two potential high-intensity subdomains of PA.

Some limitations need to be considered when interpreting our results. The identification of the subdomains household, recess at school and transport other than commuting to school was not possible. For this, a more comprehensive diary would have been needed, which would have resulted in a higher participant burden. In particular, transport-specific differences between subjects and neighborhoods require a deeper investigation in future. The overreporting of SED, LPA and MVPA in questionnaires is a common phenomenon [16]. Regarding the domain-specific PA, we cannot preclude that some children have missed reporting physical education or organized sports activities in their diary, which would have led to an underreporting of PA in these domains. Furthermore, the observed activity levels in the different domains are limited to weekdays. Finally, we need to acknowledge that our domain-specific findings are limited to children aged 6–10 years with no claim to be representative for German or European children. Future studies need to evaluate domain-specific activity levels in children older than 10 years and should investigate currently unknown factors such as the availability of playgrounds, sports facilities or safety concerns by parents that might affect the domain-specific PA levels.

## 5. Conclusions

To summarize, as about 50% of total daily SED, LPA and MVPA in young children occurred during school hours, full-time schools are a suitable setting to reduce SED and to promote PA in primary school children. Furthermore, we observed that children’s self-reported PA compared to objectively measured PA was particularly underestimated in high intensities during school hours. In order to be able to characterize the entire activity pattern in school-aged children, the application of objective measurements is compulsory.

## Figures and Tables

**Table 1:** Description of the study sample.

Sociodemographic Data	Girls (*n* = 141)	Boys (*n* = 157)	Children (6–10 Years) (*n* = 207)	Adolescents (11–17 Years) (*n* = 91)	Weekdays	Weekend Days	All (*n* = 298)
	Mean (SD)		Mean (SD)		Mean (SD)		Mean (SD)
Age (years)	10.1 (2.6)	10.5 (2.8)	8.5 (1.1)	13.6 (1.3)	-	-	10.4 (2.9)
BMI (kg/m^2^)	17.2 (3.1)	17.8 (3.8)	16.1 (2)	20.2 (4)	-	-	17.6 (3.5)
ISCED ^1^							
Low/medium (%)	24.8	26.1	20.3	34.8	-	-	25.5
High (%)	61.7	62.4	75.5	37.7	-	-	62.1
Accelerometry							
Wear-time (days)	6.1 (1)	6.1 (1.1)	6.2 (0.9)	6 (1.2)	4.5 (0.8)	1.6 (0.5)	6.1 (1)
Wear-time (min/day)	776 (53)	789 (98)	766 (71)	814 (85)	799 (80)	739 (108)	783 (80)
SED (min/day)	555 (77)	548 (116)	512 (77)	623 (96)	556 (102)	539 (117)	551 (100)
LPA (min/day)	159 (34)	166 (38)	179 (28)	134 (31)	170 (37)	145 (43)	163 (36)
MVPA (min/day)	62 (18)	74 (24)	75 (20)	56 (21)	73 (24)	55 (28)	68 (22)
>60 min MVPA (%)	52	70	76	36	69	41	62
Questionnaire							
SED (min/day)	463 (110)	463 (112)	435 (94)	523 (121)	477 (132)	439 (164)	463 (111)
LPA (min/day)	207 (81)	180 (91)	195 (88)	189 (87)	166 (91)	258 (159)	193 (87)
MVPA (min/day)	100 (81)	131 (85)	118 (78)	113 (94)	112 (81)	125 (127)	116 (84)
>60 min MVPA (%)	62	76	74	61	71	59	70

SD = standard deviation; BMI = Body Mass Index; **^1^** = 12.4% not reported; ISCED = International Standard Classification of Education; SED = sedentary behaviour; LPA = light physical activity; MVPA = moderate-to-vigorous physical activity.

**Table 2:** Average duration and proportion of self-reported and objectively measured physical activity (PA) in different domains in children (*n* = 207).

Domain	SED PAQ		SED ACC		LPA PAQ		LPA ACC		MVPA PAQ		MVPA ACC	
	Mean (SD)	% (Day)	Mean (SD)	% (Day)	Mean (SD)	% (Day)	Mean (SD)	% (Day)	Mean (SD)	% (Day)	Mean (SD)	% (Day)
Transport (min/day)	3 (9)	**1**	13 (6)	**3**	20 (13)	**11**	11 (4)	**5**	2 (7)	**1**	7 (4)	**7**
School time (min/day)	247 (114)	**52**	271 (38)	**55**	50 (41)	**27**	103 (22)	**53**	35 (39)	**20**	41 (14)	**46**
Physical education (min/week)	36 (42)	**1**	66 (44)	**2**	17 (50)	**2**	50 (28)	**4**	116 (72)	**13**	39 (23)	**8**
Leisure time (min/day)	183 (94)	**39**	182 (50)	**37**	98 (70)	**56**	56 (16)	**29**	49 (57)	**30**	21 (9)	**23**
Organized sports activities (min/week)	56 (39)	**7**	41 (36)	**4**	12 (41)	**4**	37 (27)	**9**	100 (80)	**35**	32 (28)	**16**

SED = sedentary behavior; LPA = light physical activity; MVPA = moderate-to-vigorous physical activity; PAQ = self-report; ACC = accelerometer; SD = standard deviation; min = minutes.

**Table 3:** Correlation of self-reported and objectively measured PA in children and adolescents (*n* = 298).

Age Group	SED		LPA		MVPA		>60 Min MVPA	
	r_S_	95% CI	r_S_	95% CI	r_S_	95% CI	*κ*	95% CI
Children (*n* = 207)	0.28	0.15–0.41	0.14	−0.01–0.28	0.20	0.06–0.34	0.04	−0.11–0.18
Adolescents (*n* = 91)	0.35	0.15–0.52	−0.03	−0.22–0.16	0.21	0.02–0.39	0.24	0.08–0.40
All (*n* = 298)	0.44	0.34–0.53	0.09	−0.03–0.20	0.21	0.10–0.32	0.17	0.06–0.28

SED = sedentary behaviour, LPA = light physical activity, MVPA = moderate-to-vigorous physical activity, r_S_ = Spearman coefficient, CI = confidence interval, κ = Kappa coefficient.

**Table 4:** Correlation of self-reported and objectively measured PA in different domains in children (*n* = 207).

Domain	SED		LPA		MVPA	
	r_S_	95% CI	r_S_	95% CI	r_S_	95% CI
Transport	0.24	0.11–0.37	0.59	0.49–0.67	0.12	−0.01–0.26
School time	0.18	0.04–0.31	0.14	−0.01–0.27	0.16	0.02–0.29
Physical education	0.03	−0.12–0.18	−0.03	−0.18–0.12	0.18	0.03–0.32
Leisure time	0.34	0.21–0.46	0.07	−0.07–0.21	0.12	−0.02–0.25
Organized sports activities	−0.11	−0.30–0.10	−0.01	−0.22–0.19	0.54	0.38–0.67

SED = sedentary behavior; LPA = light physical activity; MVPA = moderate-to-vigorous physical activity; r_S_ = Spearman coefficient; CI = confidence interval.

## Availability of Data

The authors do not wish to share their data, as data are not anonymized yet. Only anonymized data would be suitable for sharing.
